# Desmin Interacts Directly with Mitochondria

**DOI:** 10.3390/ijms21218122

**Published:** 2020-10-30

**Authors:** Alexander A. Dayal, Natalia V. Medvedeva, Tatiana M. Nekrasova, Sergey D. Duhalin, Alexey K. Surin, Alexander A. Minin

**Affiliations:** 1 Institute of Protein Research of Russian Academy of Sciences, Vavilova st., 34, 119334 Moscow, Russia; alexrhea9999@gmail.com (A.A.D.); medveanna@mail.ru (N.V.M.); tasha.nekrasova179@gmail.com (T.M.N.); sergey.dukhalin@phystech.edu (S.D.D.); alan@vega.protres.ru (A.K.S.); 2 Pushchino Branch, Shemyakin–Ovchinnikov Institute of Bioorganic Chemistry, Russian Academy of Sciences, Prospekt Nauki 6, Pushchino, 142290 Moscow Region, Russia

**Keywords:** mitochondria, desmin, intermediate filaments, calpain

## Abstract

Desmin intermediate filaments (IFs) play an important role in maintaining the structural and functional integrity of muscle cells. They connect contractile myofibrils to plasma membrane, nuclei, and mitochondria. Disturbance of their network due to desmin mutations or deficiency leads to an infringement of myofibril organization and to a deterioration of mitochondrial distribution, morphology, and functions. The nature of the interaction of desmin IFs with mitochondria is not clear. To elucidate the possibility that desmin can directly bind to mitochondria, we have undertaken the study of their interaction in vitro. Using desmin mutant Des(Y122L) that forms unit-length filaments (ULFs) but is incapable of forming long filaments and, therefore, could be effectively separated from mitochondria by centrifugation through sucrose gradient, we probed the interaction of recombinant human desmin with mitochondria isolated from rat liver. Our data show that desmin can directly bind to mitochondria, and this binding depends on its N-terminal domain. We have found that mitochondrial cysteine protease can disrupt this interaction by cleavage of desmin at its N-terminus.

## 1. Introduction

**Figure 1:**
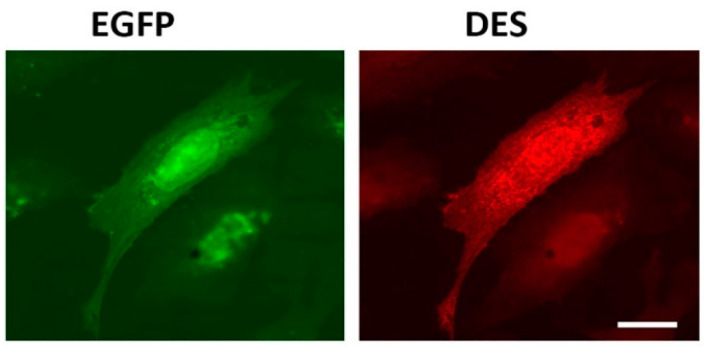
Immunofluorescence of REF-52(Vim^−/−^) cells transfected with plasmid pIRES-EGFP-Des(Y122L). Expression of EGFP (left) and desmin(Y122L) (right) in rat fibroblast stained with antibodies against desmin shows evenly distributed unit-length filaments (ULFs) but not long intermediate filament (IF). Scale 10 µm.

Intermediate filament (IF) networks are one of three cytoskeletal components along with microtubules and actin microfilaments. IFs provide mechanical strength and elasticity to cells and tissues and regulate intracellular positioning of nucleus and organelles [1]. It has been shown that one of the important roles of IFs inside animal cells is to ensure normal mitochondrial functioning [2,3,4,5,6,7]. Desmin, a major constituent of the IF network inside muscle cells, is a good example of such factors controlling mitochondria. A plethora of pathological conditions, collectively known as desminopathies, is caused by mutations in desmin and results in abnormalities in mitochondrial distribution and morphology as well as reduced mitochondrial respiratory function [5,7,8,9]. These data suggest that interaction with desmin is a prerequisite for normal mitochondrial functioning. However, the nature of interaction between desmin IFs and mitochondria is not fully elucidated. For example, one question that needs to be asked is whether desmin is able to bind directly to these organelles or some intermediary proteins are necessary. Previously, in G. Wiche’s lab, it was demonstrated that one such intermediary is plectin, particularly its 1b isoform, which interacts both with desmin and mitochondria [10]. However, experimental evidence showed certain desmin mutations which do not interfere with native IF structure or apparently with binding of plectin yet cause pathological conditions [11], including mitochondrial dysfunction [7]. A direct association between desmin IFs and mitochondria could explain these phenomena. In this study, we examined the possibility of a direct interaction between purified recombinant desmin and isolated rat liver mitochondria. We performed centrifugation in the sucrose gradient to separate desmin and a heavier mitochondrial fraction. To prevent sedimentation of unbound desmin, we used its mutant form Des(Y122L). This point mutation in desmin arrests its polymerization at the stage of unit-length filaments (ULFs) consisting of approximately 30–40 polypeptides of desmin that are much lighter than long IFs and sediment only at high-speed centrifugation. Figure 1 shows that Des(Y122L) when expressed in rat fibroblasts REF-52(Vim^−/−^) devoid of IFs is unable to form filaments. It rather forms ULFs evenly distributed throughout the cytoplasm, similarly to mutant vimentin(Y117L) [12,13]. 

Our results demonstrate that desmin in the form of ULFs binds to mitochondria directly, and the N-terminus of its molecule is responsible for this interaction. 

## 2. Results

### 2.1. Desmin Binds to Mitochondria In Vitro

Previously, we hypothesized [6] that, similarly to vimentin, an N-terminal part of desmin molecule could contain a targeting signal which localizes desmin to the outer mitochondrial membrane (OMM). Its moderately hydrophobic region spanning from threonine-17 to proline-36 is flanked by two groups of positively charged amino acids—a characteristic pattern of many proteins known to be localized to OMM [14]. To predict the presence of mitochondrial targeting peptide in the molecule of human desmin, we used the online program TargetP 1.1 [15] to analyze its sequence and found that such signal is present with a high probability (mTP—0.864). Therefore, according to the analysis of desmin’s primary structure, it belongs to the proteins predisposed to localize in mitochondria.

To test the possibility of the direct binding of desmin to mitochondria, we probed the interaction of mitochondria from a rat liver with a recombinant human Des(Y122L) in vitro. The protein was expressed and purified from bacterial lysate (see Supplementary Methods and Figure S1A). The bound Des(Y122L) was determined using Western blotting of mitochondrial pellets obtained by centrifugation of their mixtures through sucrose cushion. 

**Figure 2:**
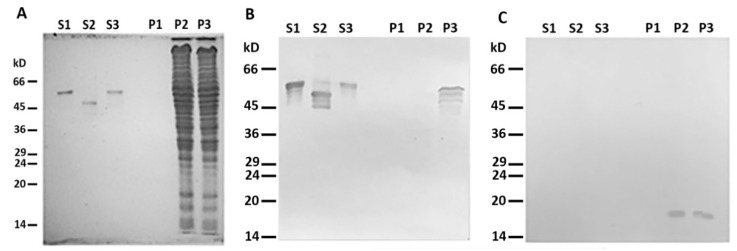
Desmin and mitochondria co-sedimentation. (**A**) SDS-PAGE of supernatants (S1, S2, S3) and pellets (P1, P2, P3) after centrifugation through sucrose cushion of mixtures of Des(Y122L) and mitochondria. Des(Y122L) was incubated without mitochondria (S1, P1), with mitochondria (S2, P2), or with mitochondria and protease inhibitor cocktail (S3, P3). Western blotting of the same samples as in (**A**) with antibodies against desmin or COX-IV is shown in (**B**,**C**), respectively. The desmin molecule approximately 10 kD smaller after the incubation with mitochondria (S2) does not co-sediment with mitochondria (P2), while the addition of protease inhibitor cocktail prevents desmin cleavage (S3) and ensures its co-sedimentation with mitochondria (P3). COX-IV is detectable only in lines P2 and P3 which shows the presence of mitochondrial proteins in the pellets.

When centrifuged through a sucrose cushion, such desmin mutant did not sediment by itself and completely stayed in the supernatant (Figure 2A,B, lines S1 and P1). However, when the sample contained mitochondria, some Des(Y122L) was found in the pellet, but only in the case where the protease inhibitor cocktail was present in the mixture (Figure 2A,B, lines S3 and P3). Incubation of this protein with mitochondria without protease inhibitors led to partial protein degradation, and the shortened Des(Y122L) was found only in the supernatant (Figure 2A,B, lines S2 and P2). COX-IV was used as a marker of mitochondrial proteins in the pellets and the loading control (Figure 2C). Hence, full-size Des(Y122L) bound to mitochondria and co-sedimented with them through sucrose cushion, while cleaved desmin did not. 

**Figure 3:**
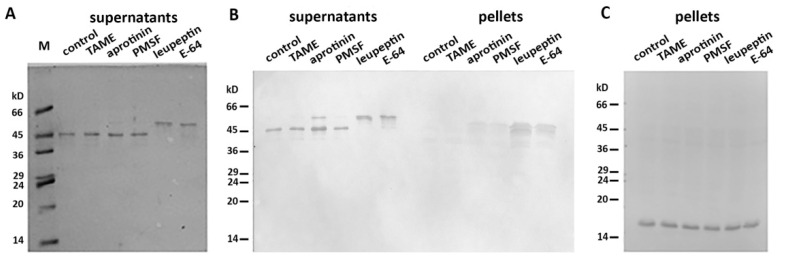
Inhibitory analysis of desmin degradation. Protease inhibitors are indicated. (**A**) Samples stained with Coomassie Brilliant Blue after electrophoresis in 15% SDS-PAGE of desmin and mitochondria mixtures after centrifugation through sucrose cushion. Only leupeptin and E-64 inhibitors prevent the formation of 45 kD desmin cleavage product. (**B**) Immunoblotting of desmin and mitochondria mixtures after centrifugation through sucrose cushion. Only intact desmin molecule co-sediments with mitochondria (leupeptin and E-64 lines in pellets). (**C**) Immunoblotting of COX-IV in the pellet fractions of the samples. COX-IV is detectable in all lines which show the presence of mitochondria.

More than 40 mitochondrial proteases are known to maintain normal functioning of these organelles [16]. We proposed that one (or several) of these mito-proteases may cleave Des(Y122L) when it interacts with mitochondria. To discover a candidate protease, we performed an inhibitory analysis. We examined several protease inhibitors, including PMSF (phenylmethylsulfonyl fluoride), aprotinin, TAME (Tosyl-L-Arginine Methyl Ester), leupeptin, and E-64, and found that only the last two inhibited desmin degradation (Figure 3). Since leupeptin and E-64 are known to inhibit cysteine proteases, in contrast to serine protease inhibitors PMSF, aprotinin, and TAME, we inferred that a candidate belongs to the cysteine proteases. Figure 3B shows that protection of Des(Y122L) against cysteine proteases allows its co-sedimentation with mitochondria. 

**Figure 4:**
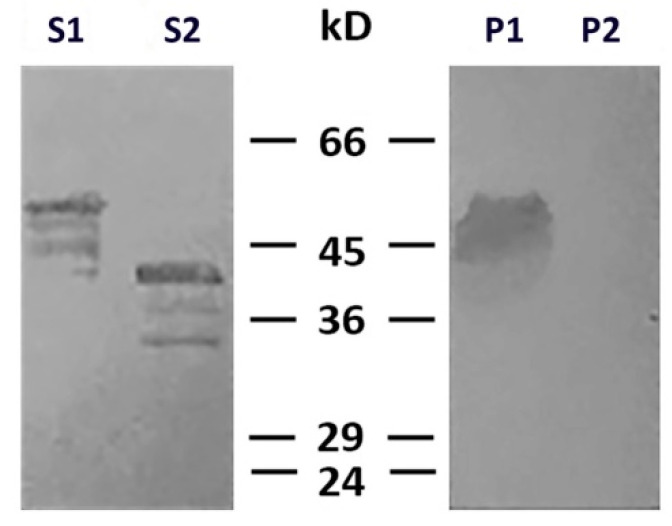
Effects of calpeptin and PD150606 on desmin and mitochondria co-sedimentation. Immunoblotting of desmin in supernatants (S1, S2) and pellets (P1, P2) after centrifugation through sucrose cushion. Desmin(Y122L) was incubated with mitochondria and calpeptin (S1, P1) or PD150606 (S2, P2). Non-cleaved desmin is seen both in supernatant and pellet only when calpeptin was added (S1, P1). Allosteric inhibitor PD150606 did not protect desmin from proteolysis (S2, P2).

To determine which type of cysteine protease was involved in desmin degradation, we used more specific inhibitors—calpeptin and PD150606. It turned out that calpeptin, which inhibits calpains and cathepsins L and K [17,18], effectively protected desmin (Figure 4, lines S1 and P1), while PD150606, which specifically inhibits calpains I and II [19], did not prevent protein degradation (Figure 4, lines S2 and P2). Since PD150606 inhibits only typical calpains that possess domain IV, we proposed that the protease responsible for desmin cleavage is atypical calpain-10, which has been found in mitochondria earlier [20]. Although the contamination of mitochondria with lysosomal cathepsins cannot be completely ruled out, their participation is unlikely since available data on degradation of desmin by cathepsin [21] show no clear electrophoretic fragments but rather smear-like pattern. 

### 2.2. N-terminus of Desmin is Necessary for Binding to Mitochondria

N- and C-termini of the desmin molecule are the most protease-sensitive regions, whereas the central alpha-helical domain is relatively stable [10,21]. Our data (Figure 2, Figure 3 and Figure 4) show that the main product of desmin degradation resulting from the incubation with mitochondria was smaller than the initial polypeptide by approximately 10 kD. To identify which part of desmin molecule is cleaved, we performed mass-spectrometry analysis of the protein band in the gel corresponding to the cleavage product. The data demonstrated that desmin molecule was full-length when protected by leupeptin (Figure S1A) but lost its 70 amino acid-long fragment from N-terminus when incubated with mitochondria in the absence of inhibitors (Figure S1B). Hence, the cleavage of N-terminus might be responsible for the loss of ability of desmin to interact with mitochondria. Interestingly, the analysis of such shortened desmin using TargetP 1.1 showed very low probability of a mitochondrial target peptide in it (mTP—0.122). 

**Figure 5:**
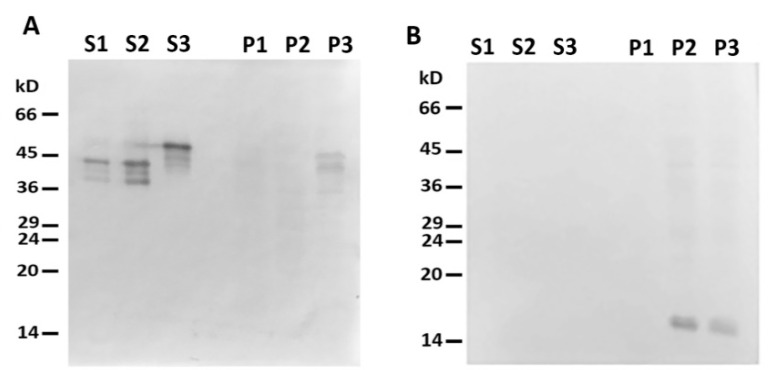
Headless ΔN-Des(Y122L) does not bind to mitochondria. (**A**) Western blotting of supernatants (S1, S2, S3) and pellets (P1, P2, P3) after centrifugation through sucrose cushion of mixtures of ΔN-Des(Y122L) (S2, P2) or Des(Y122L) (S3, P3) and mitochondria. The truncated protein ΔN-Des(Y122L) does not sediment without mitochondria (S1, P1). (**B**) Western blotting of the same samples as in **A** with antibodies against COX-IV shows the loading control of mitochondrial pellets.

To further elucidate the role of the N-terminal domain of desmin in its binding to mitochondria, we probed the interaction of the headless ΔN-Des(Y122L) obtained by the aforementioned procedure, including the incubation with mitochondria and subsequent centrifugation through sucrose gradient. We preferred such an approach to the expression of shortened polypeptide in bacteria because the formation of the dimers of type III IFs as well as the subsequent steps leading to generation of ULFs is strongly dependent on the intact N-terminal domain [22]. The analysis of the truncated ΔN-Des(Y122L) from the supernatants after centrifugation using SDS-PAGE showed that it was pure enough and in sufficient amount (Figure 2A and Figure 3A). The data in Figure 5A demonstrate that ΔN-Des(Y122L) lacking N-terminus does not co-sediment with mitochondria in contrast to the entire non-truncated molecule that was treated similarly but in the presence of protease inhibitor leupeptin. Thus, the loss of N-terminal fragment of desmin molecule disrupts its binding to mitochondria. 

**Figure 6:**
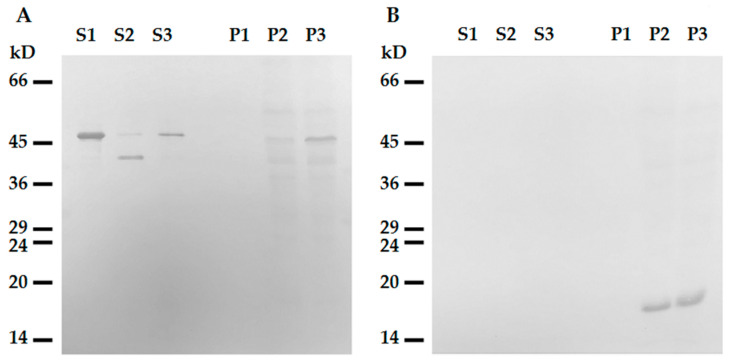
Interaction of tailless desmin and mitochondria. (**A**) Western blotting of ΔC-Des(Y122L) in supernatants (S1, S2, S3) and pellets (P1, P2, P3) after centrifugation through sucrose cushion. ΔC-Des(Y122L) was incubated 20 min without mitochondria (S1, P1), with mitochondria but without protease inhibitors (S2, P2), or with mitochondria and leupeptin (S3, P3). (**B**) Western blotting of COX-IV in the same samples shows loading control in mitochondrial pellets.

To rule out the possibility of participation of the C-terminal domain in this interaction, we constructed the deletion mutant of desmin with the truncated C-terminal region 53 amino acids long. This ΔC-Des(Y122L) mutant also containing the point mutation Y122L was expressed in bacteria and purified similarly to the original protein (see Supplementary Methods and Figure S1B). Our data show that desmin missing C-terminus bound to mitochondria when its N-terminal part was protected from proteolysis by leupeptin (Figure 6, line P3). However, incubation in the absence of inhibitor led to its shortening and to the loss of the ability to co-sediment with mitochondria (Figure 5, lines S2 and P2). This protein did not sediment without mitochondria in these conditions (Figure 5, lines S1 and P1). The analysis of ΔC-Des(Y122L) by mass-spectrometry in the presence (Figure S1C) or in the absence of leupeptin (Figure S1D) showed that proteolysis led to the cleavage of the identical fragment (70 amino acids) to that of the whole Des(Y122L). Thus, N-terminal domain of desmin is necessary for the interaction with mitochondria, while C-terminus is unimportant. 

## 3. Discussion

Our results show that pure recombinant Des(Y122L) can bind to isolated rat liver mitochondria without any intermediary proteins. More specifically, desmin uses its N-terminal domain for interaction with mitochondria. These data are in good agreement with the results of analysis of desmin polypeptide with TargerP 1.1, which predict mitochondrial targeting signal in its N-terminus. Such signals are recognized by special protein complexes TOM and TIM localized in the outer and inner mitochondrial membranes, respectively. These complexes are responsible for translocation of proteins carrying targeting signals and participate in their subsequent distribution into different mitochondrial compartments [23]. However, desmin, similarly to other IF proteins, forms large polymeric structures, the smallest of which, tetramers, contain a coiled-coil core structure of four polypeptides. Thus, upon the binding of the targeting peptide to the TOM complex, further translocation may be impeded due to the large sizes of these particles. This is true for ULFs formed by Des(Y122L) in our experiments. It suggests that interaction of desmin filaments with mitochondria can depend on the binding of its N-terminus to the mitochondrial import complex. Whether such interaction is common to other IF proteins is an intriguing question to be examined in future studies. Nevertheless, our previous experiments that involved vimentin IFs [6,13] argue in favor of universality of such mechanism. 

The presence of mitochondrial targeting peptide in the N-terminus of desmin could have one more important implication—it is this part of the molecule that appears inside the mitochondrion, whereas central and C-terminal domains reside in the cytoplasm. This is indirectly confirmed by the N-terminus cleavage occurring in the first place. According to our inhibitory analysis, the most probable mito-protease responsible for this cleavage is atypical calpain-10, which was detected in mitochondrial matrix and intermembrane space [20]. An additional argument in favor of the participation of calpain is the cut site at arginine-70 of desmin, which is revealed in the product of proteolysis by mass-spectroscopy (Figure S2B,D). This site in the desmin polypeptide is predicted for calpains by the DeepCalpain web server (http://deepcalpain.cancerbio.info/index.php) based on the deep learning method [24]. Importantly, lysosomal cathepsin activity, which can also be inhibited by calpeptin [17,18] and could be present in mitochondria preparations as a contamination, would have likely resulted in C-terminus cleavage. However, we did not observe it in our experiments. Thus, calpain-10 being a Ca^2+^-dependent protease can be involved in regulation of the binding of mitochondria to desmin IFs by Ca^2+^ level, which is high in the mitochondrial matrix and low in the intermembrane space and in cytosol. Depolarization of the inner mitochondrial membrane causing the Ca^2+^ efflux would lead to calpain activation and as a result to the cleavage of desmin’s N-terminus, which is responsible for the interaction. Therefore, the binding of mitochondria to desmin IFs could be dependent on their membrane potential, and the disruption of this bond by calpain-10 due to a decrease of potential might play a role in the quality control, though it should be verified directly in future studies. 

## 4. Materials and Methods

### 4.1. Cell Culture, Plasmids and Antibodies

The cells REF-52(Vim^−/−^) that were generated earlier using the CRISPR-Cas9 system [25] were cultured in DMEM (Dulbecco’s Modified Eagle Medium) with 10% fetal bovine serum containing 100 µg/mL penicillin and 100 µg/mL streptomycin at 37 °C in the humid atmosphere with 5% CO_2_. 

For the expression of recombinant desmin in bacteria, we used pET-23b(+) vector. First, to make the intermediary plasmid pET-23b(+)-Desm, we amplified cDNA encoding human desmin (Addgene, IMAGE:4905678) by PCR using primers TTTCATATGAGCCAGGCCTACTCG and TTTAAGCTTTTAGAGCACTTCATGCTGCT and inserted it into the vector at sites Nde I and Hind III. The plasmid pET-23b(+)-Desm (Y122L) encoding desmin with the substitution of tyrosine-122 to leucine was generated using the inverse PCR [26] of the plasmid pET-23b(+)-Desm with primers TTAATCGAGAAGGTGCGCTTCCTGG and GTTGGCGAAGCGGTCATTGAGC and the ligation of its blunt ends. The plasmid pET-23b(+)-(deltaC)-Desm(Y122L) encoding desmin, missing the 53 amino acids at its C-terminus, was generated also using the inverse PCR of the plasmid pET-23b(+)-Desm(Y122L) with the primers TTAATCGAGAAGGTGCGCTTCCTGG and GTTGGCGAAGCGGTCATTGAGC followed by the ligation of product’s blunt ends. 

pIRES-EGFP-Des(Y122L) plasmid was constructed via standard cloning into the corresponding vector in the EcoR1 site by using cDNA from pET-23b(+)-Desm(Y122L) plasmid. The accuracy of the products was checked by sequencing. The transfection of the cells REF-52(Vim^−/−^) with plasmid pIRES-EGFP-Desm(Y122L) was performed using TransIT-LT1 reagent (Mirus Bio, Madison, WI, USA) according to the instruction. 

Monoclonal mouse antibodies DE-U-10 against desmin (Sigma, St. Louis, MO, USA), kindly gifted by Dr. G. Agnetti (Baltimore, MD, USA), were used for Western blotting and for immunofluorescence; and monoclonal rabbit antibodies 3E11 against COX-IV (Cell Signalling, Danvers, MA, USA) for Western blotting. As secondary antibodies, we used TRITC-labeled goat anti-mouse antibodies, and horseradish peroxidase-linked anti-mouse and anti-rabbit IgG, (The Jackson Laboratory, Bar Harbor, ME, USA). 

### 4.2. Transfection of Cells and Immunofluorescence

Transfection of REF-52(Vim^−/−^) cells with plasmid pIRES-EGFP-Des(Y122L) was performed using TransIT-LT1 Transfection Reagent (Mirus Bio, USA). DNA (1 mg) was used for a 40 mm plate of cells containing 2 mL of a culture medium. A total of 16–20 h after transfection, cells were fixed with 4% formaldehyde in PBS followed by permeabilization with 0.1% Triton X-100, and stained with anti-desmin antibodies DE-U-10 and secondary TRITC-labeled goat anti-mouse antibodies as previously described [6]. Microscopy was carried out with a Keyence BZ-9000 (USA) equipped with a PlanApo 63x, 1.4 NA objective. Images were captured with inbuilt 12-bit camera and processed ImageJ software. The experiment was performed 3 times.

### 4.3. Isolation of Mitochondria

Mitochondria were isolated from rat liver. Animals were maintained in compliance with regulations on the use and care of laboratory animals. The liver was immediately removed and put into ice-cold PBS, cut to small pieces with scissors, and washed. Homogenization was performed in Potter homogenizer in isolation buffer—10 mM K-Hepes pH 7.4 with 220 mM mannitol, 70 mM sucrose, 1 mM EDTA). Homogenate was centrifuged at 3000 rpm for 10 min in a JA-20 rotor. The supernatant was filtered through the gauze and then centrifuged at 9500 rpm for 20 min in a JA-20 rotor. The mitochondria pellet was homogenized in isolation buffer and then centrifuged at 9500 rpm for 20 min in a JA-20 rotor. The pellet was re-suspended in minimal volume of isolation buffer (1 mL total) and layered onto sucrose gradient which consisted of 1.2 M sucrose and 1.6 M sucrose in 10 mM K-Hepes buffer (pH 7.4) with 1 mM of EDTA and centrifuged at 30,000 rpm for 20 min in a TLS-55 rotor. Brownish fraction containing mitochondria, clearly distinguishable at the interphase between the two sucrose layers, was carefully collected, diluted with isolation buffer, and pelleted at 9500 rpm for 10 min in JA-21 rotor. The pellet was re-suspended minimal volume of isolation buffer and contained 60 ± 20 mg/mL of mitochondrial protein. 

### 4.4. Interaction of Mitochondria with Desmin

To determine the interaction of mitochondria with Des(Y122L), we used the centrifugation through a sucrose cushion. Desmin bound to mitochondria appeared in the pellet, while an unbound protein stayed in supernatant. Incubation mixture (total volume of 480 µL) contained 0.1 mg/mL of desmin and 2 mg/mL of mitochondria in 10 mM K-Hepes buffer pH 7.4 with 220 mM mannitol, 70 mM sucrose, and 1 mM EDTA. Final concentrations of protease inhibitors when mentioned were: PMSF—0.35 mg/mL; aprotinin—1 µg/mL; TAME—1 µg/mL; E-64—10 µg/mL; leupeptin—1 µg/mL; calpeptin—15 µg/mL; PD150606—20 µg/mL. After incubation at 25 °C for 20 min, 400 µL of each sample was layered on 500 µL of sucrose cushion (1.2 M sucrose, 10 mM K-Hepes pH 7.4, and 1 mM EDTA) and centrifuged in a TLS-55 rotor at 30,000 rpm for 10 min. A total of 200 µL of supernatant (upper phase), containing unbound desmin, was collected for analysis or further usage in binding experiments. The sucrose cushion was carefully washed with distilled water and removed by aspiration. The mitochondrial pellet was re-suspended in 100 µL of water. Samples of supernatants and the pellets were mixed with Laemmli sample buffer, incubated for 10 min at 98 °C, and separated using 15% SDS-PAGE. Protein bands in gels were revealed by Coomassi Brilliant Blue staining or transferred to nitrocellulose membrane for subsequent Western blot analysis with anti-desmin [1:1000] and anti-COX-IV antibodies [1:1000], and secondary HRP-conjugated antibodies [1:2000], using 3,3′-Diaminobenzidine as a substratum. Each experiment was performed at least 3 times. 

The exact same procedure was used to determine the interaction of mitochondria with ΔC-Des(Y122L). To study the binding of ΔN-Des(Y122L) to mitochondria, we used the truncated protein provided by incubation of Des(Y122L) with mitochondria in the absence of protease inhibitors. For that, we collected supernatant containing unbound desmin after centrifugation, added leupeptin to avoid further protein degradation, and mixed with a fresh portion of mitochondria. To make a control with the full-length Des(Y122L), we added leupeptin during the first incubation with mitochondria and used supernatant containing non-truncated protein for the second incubation with mitochondria. 

### 4.5. Detection of Mitochondrial Targeting Signals

To detect possible mitochondrial targeting signal, we used the tool TargetP 1.1, available online (http://www.cbs.dtu.dk/services/TargetP-1.1/index.php). This method uses two-layer artificial neural networks trained on UniProt annotated sequences with experimentally established localization signals [15]. The result of TargetP 1.1 analysis is an mTP score which predicts the presence of mitochondrial targeting peptide in sequences of proteins. The value of mTP that exceeds 0.55 indicates a high probability of the presence of such signal. 

## Supplementary Materials

The following are available online at https://www.mdpi.com/1422-0067/21/21/8122/s1. Supplementary Methods: purification of desmin; mass-spectrometry. Supplementary Figures: Figure S1. Expression and purification of recombinant desmin variants in bacteria. SDS-PAGE of (A) Des(Y122) and (B) ΔC-Des(Y122L) samples at the steps of purification. 1—homogenates; 2—supernatants obtained after the sedimentation of inclusion bodies; 3—inclusion bodies pellets; 4—inclusion bodies after washing with 1% Triton X-100; 5—inclusion bodies after washing with 1 M NaCl; Figure S2. Mass-spectroscopy of Des(Y122L) and ΔC-Des(Y122L) after incubation with mitochondria. The protein bands in gels corresponding to Des(Y122L) (A) before and (B) after incubation with mitochondria in the absence of protease inhibitors were processed for analysis as described in Supplemental Methods. C, D—similar results obtained for the shortened protein ΔC-Des(Y122L). Revealed peptides are shown as red rectangles superimposed on the sequence of the human desmin.
