# Supplementary material for: Desmin Interacts Directly with Mitochondria
Source: Int J Mol Sci. 2020 Oct 30;21(21):8122. doi: 10.3390/ijms21218122 (PMC7663591; doi:10.3390/ijms21218122)
Supplement: Supplementary file 1 [file ijms-21-08122-s001.pdf]

International Journal of
Molecular Sciences

MDPI

SUPPLEMENTARY MATERIALS.

Supplementary Methods.

Purification of desmin.

Proteins were expressed in *E. coli* BL21 cells, transformed with the corresponding plasmid. Bacteria were incubated in LB medium at 37 °C overnight. A total of 25 mL of a resulting culture was then transferred in 500 mL of 3x LB medium supplemented with carbenicillin (100 µg per mL) and incubated at 37 °C for a day with an intense stirring and then left growing at 30 °C overnight. The resultant cell suspension was centrifuged at 4000 g for 20 min, and the pellet was re-suspended in 50 mL of lysis-buffer 5 mM Tris-HCl pH 8.4 with 1 mM EDTA, 1 mM EGTA, and protease inhibitor cocktail (S8830, Sigma, USA). The cells in the resultant mixture were kept frozen at –20 °C. The thawed cells were incubated with lysozyme for 30 min on ice and then sonicated for 4 min using ultrasonic disintegrator Sonic Dismembrator 550 (Fisher Scientific, USA). The homogenate was centrifuged for 30 min at 15000 rpm in a JA-20 rotor (Beckman, USA), and the pellet containing inclusion bodies was washed successively in the lysis-buffer, in the lysis-buffer supplemented with 1% Triton X-100, the lysis buffer containing 1 M NaCl, and the lysis-buffer alone. The resulting suspensions contained pure Des(Y122L) and ΔC-Des(Y122L) (Figure S1A,B, respectively) and was kept frozen at –20 °C. The desmin in the inclusion bodies was first dissolved using 8 M urea in 5 mM Tris-HCl buffer (pH 8.4) containing a protease cocktail inhibitor (S8830, Sigma) and centrifuged for 30 min at 15,000 rpm in a JA-20 rotor. Then, protein renaturation was performed using dialysis against 5 mM Tris-HCl buffer (pH 8.4) with gradually decreasing concentration of urea: 6 M for 30 min at room temperature, 4 M for 30 min at room temperature, 2 M for 30 min at 4 °C, and in buffer without urea at 4 °C twice for 60 min. The final protein solution was centrifuged at 30,000 rpm for 30 min in a TLS-55 rotor, and the protein concentration was measured via the BCA method (usually approx. 2.0 mg per mL).

Mass-spectrometry.

For mass-spectrometry, protein bands were cut from gels stained with Coomassie and sequentially washed with 40% methanol and 5% acetic acid solution for 15 min per detergent, and with 50% acetonitrile in 50 mM NH_4HCO_3 for 20 min at 56 °C to remove dye. For the alkylation of SH-groups, gel pieces were then incubated in 5 mM DTT solution for 30 min and in 15 mM iodoacetamide for 30 min.

Before incubation in trypsin, chymotrypsin and proteinase K solution gel pieces were dehydrated by acetonitrile and dried up using a vacuum concentrator 5301 (Eppendorf, Germany) for 5 min. Excess amount of enzymes was removed, and gels were incubated in 100 µL of 0.1 M NH_4HCO_3 at 37 °C for 20 hours while shaking. Gel extracts, obtained through incubation in 80% acetonitrile and 0.1% TFA at 25 °C for 30 min, were combined with peptide eluates and dried up in a vacuum concentrator. The analysis of samples was performed on a high-resolution mass-spectrometer OrbiTrap Elite (Thermo Scientific, Germany). Protein hydrolysates were separated on a liquid chromatography system Easy nLC 1000 (Thermo Scientific, USA) on the column 150 mm x 75 µm filled with reverse phase C18 (3 µm particle size and 100Å pore size) prepared in laboratory settings. The following eluates were used: A) 3% acetonitrile in deionized water with 0.05% of formic acid, and B) 80% acetonitrile in deionized water with 0.05% of formic acid. Panoramic mass spectra and fragmentation spectra were recorded with resolutions of 60,000 and 15,000, respectively. The fragmentation of ions was performed using the collision-induced dissociation technique. Data analysis was performed in commercially available software package PEAKS Studio 7.5.

Supplementary Figures.

Figure S1.

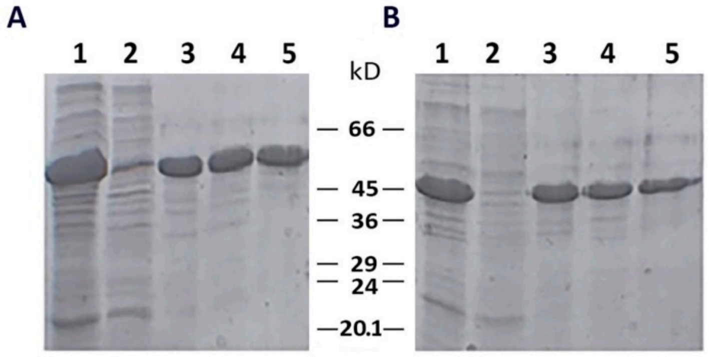

Figure S2 (A).

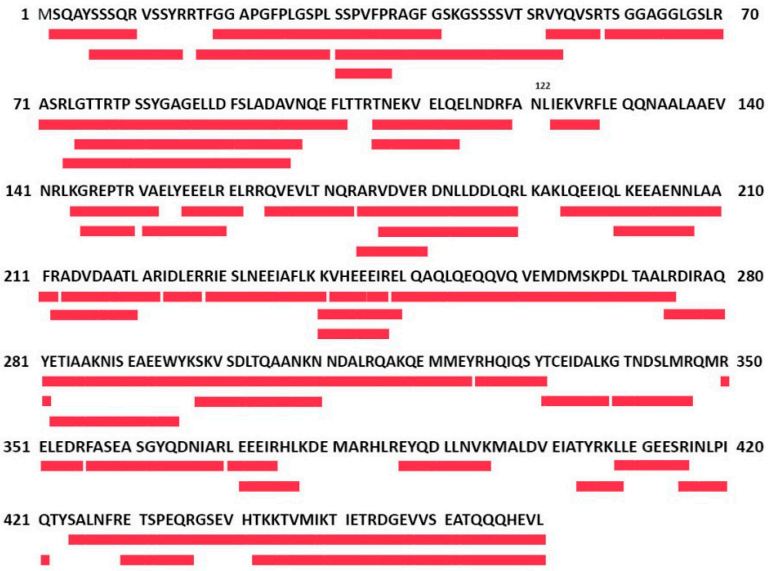

Figure S2 (B).

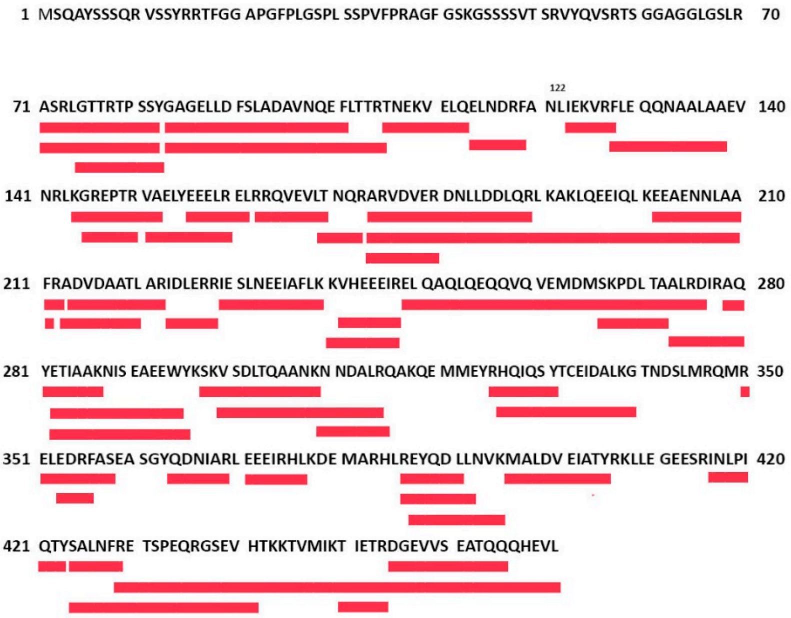

Figure S2 (C).

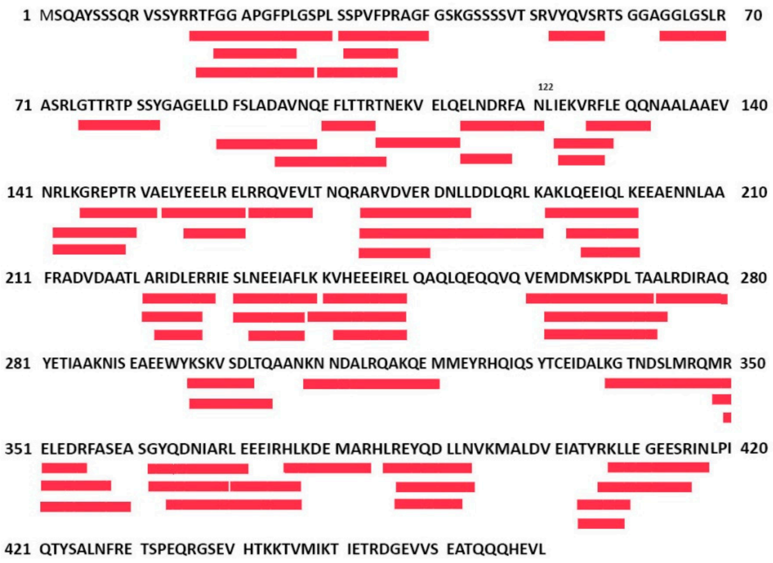

Figure S2 (D).

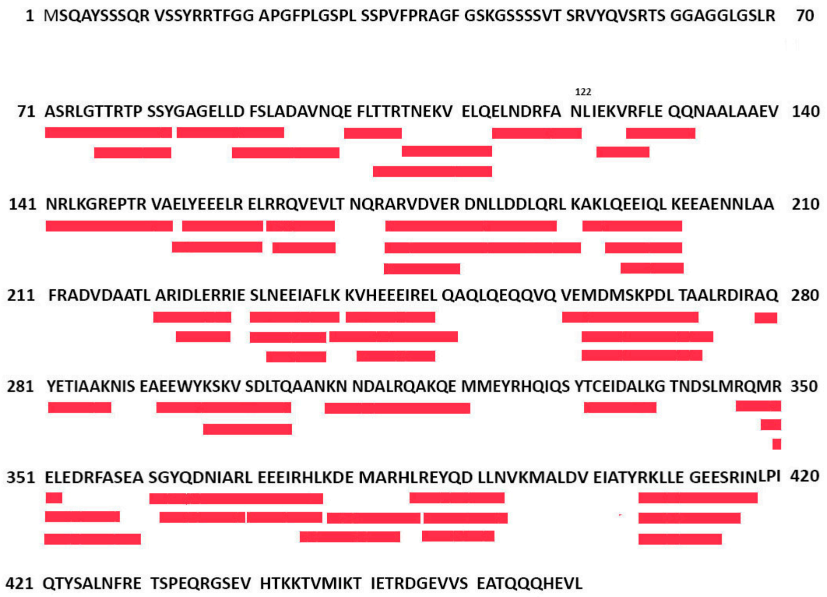
1 MSQAYSSSQR VSSYRRTFGG APGFPLGSPL SSPVFPRAGF GSKGSSSSVT SRVYQVSRTS GGAGGLGSLR 70
122
71 ASRLGTTRTP SSYGAGELLD FSLADAVNQE FLTTRTNEKV ELQELNDRFA NLIEKVRFLE QQNAALAAEV 140
141 NRLKGREPTR VAELYEEELR ELRRQVEVLT NQRARVDVER DNLLDDLQRL KAKLQEEIQL KEEAENNLAA 210
211 FRADVDAATL ARIDLERRIE SLNEEIAFLK KVHEEEIREL QAQLQEQQVQ VEMDMSKPDL TAALRDIRAQ 280
281 YETIAAKNIS EAEEWYKSKV SDLTQAANKN NDALRQAKQE MMEYRHQIQS YTCEIDALKG TNDSLMRQMR 350
351 ELEDRFASEA SGYQDNIARL EEEIRHLKDE MARHLREYQD LLNVKMALDV EIATYRKLLE GEESRINLPI 420
421 QTYSALNFRE TSPEQRGSEV HTKKTVMIKT IETRDGEVVS EATQQQHEVL
